# Exploring Chronic Pain and Pain Management Perspectives: Qualitative Pilot Analysis of Web-Based Health Community Posts

**DOI:** 10.2196/41672

**Published:** 2023-05-30

**Authors:** Claire Harter, Marina Ness, Aleah Goldin, Christine Lee, Christine Merenda, Anne Riberdy, Anindita Saha, Richardae Araojo, Michelle Tarver

**Affiliations:** 1 Inspire Arlington, VA United States; 2 US Food and Drug Administration Silver Spring, MD United States

**Keywords:** chronic pain, pain management, online health community

## Abstract

**Background:**

Patient perspectives are central to the US Food and Drug Administration’s benefit-risk decision-making process in the evaluation of medical products. Traditional channels of communication may not be feasible for all patients and consumers. Social media websites have increasingly been recognized by researchers as a means to gain insights into patients’ views about treatment and diagnostic options, the health care system, and their experiences living with their conditions. Consideration of multiple patient perspective data sources offers the Food and Drug Administration the opportunity to capture diverse patient voices and experiences with chronic pain.

**Objective:**

This pilot study explores posts from a web-based patient platform to gain insights into the key challenges and barriers to treatment faced by patients with chronic pain and their caregivers.

**Methods:**

This research compiles and analyzes unstructured patient data to draw out the key themes. To extract relevant posts for this study, predefined keywords were identified. Harvested posts were published between January 1, 2017, and October 22, 2019, and had to include #ChronicPain and at least one other relevant disease tag, a relevant chronic pain management tag, or a chronic pain management tag for a treatment or activity specific to chronic pain.

**Results:**

The most common topics discussed among persons living with chronic pain were related to disease burden, the need for support, advocacy, and proper diagnosis. Patients’ discussions focused on the negative impact chronic pain had on their emotions, playing sports, or exercising, work and school, sleep, social life, and other activities of daily life. The 2 most frequently discussed treatments were opioids or narcotics and devices such as transcutaneous electrical nerve stimulation machines and spinal cord stimulators.

**Conclusions:**

Social listening data may provide valuable insights into patients’ and caregivers’ perspectives, preferences, and unmet needs, especially when conditions may be highly stigmatized.

## Introduction

Patients are at the heart of what the US Food and Drug Administration (FDA) does and are vital to the agency’s work of protecting public health by ensuring the safety and efficacy of drugs, biological products, and medical devices [1]. Understanding patient perspectives can aid the agency in numerous ways; review staff can better understand patient experience, consider symptom management and side effects, impact of treatment on quality of life, and risk-benefit profiles.

Traditionally, the patient voice is heard through channels such as participation at formal meetings, letters to the agency, docket comments, or survey responses. It is important to recognize that not all patients are familiar or comfortable with using traditional ways of communicating with organizations such as the FDA. Confounded with this barrier are the unique challenges that racial and ethnic minorities and underserved populations encounter, such as mistrust [2]. Medical mistrust can hinder communication and sharing of information such as medical history and patient experiences [3]. This reticence to share experiences is often further amplified in discussions of stigmatized disease conditions such as chronic pain [4].

Over the past few decades, advances in technology have enabled researchers and health care providers to gain insights into patients’ perspectives in ways that have not been previously possible. Social media websites have been increasingly recognized as a platform for patients to gather information, explore options, and share their experiences [5]. With over 80% of Americans seeking and sharing health information online through blogs, microblogging (eg, Twitter), social networking (eg, Facebook), and video and file-sharing sites (eg, YouTube), social media cannot be ignored [6,7]. Social listening is one potential avenue that can be leveraged to gain insights into the patient experience.

Incorporation of the patient voice is an important aspect of regulatory decision-making, supported by the 21st Century Cures Act (Cures Act). The Cures Act builds on the FDA’s ongoing work to incorporate patients’ perspectives into the development of regulated products and regulatory decision-making process [8].

The Center for Devices and Radiological Health aims to ensure patients are at the center of its regulatory decision-making process. It does this through encouraging patient engagement, the incorporation of clinical outcome assessments in medical device clinical investigations, and the collection of patient preference information [9]. The FDA Office of Minority Health and Health Equity (OMHHE) also supports efforts to amplify equity of voices through its Enhance Equity Initiative by (1) expanding OMHHE’s diverse stakeholder community, (2) supporting research to leverage novel and big data sources to understand diverse patient perspectives, preferences, and unmet needs, and (3) supporting expansion of culturally and linguistically tailored health education [10].

Many internet users seek health information through online health communities and other social media, including sharing information and well as finding value in peer-generated health information [11-13]. This study supports the priority, Empowering Patients and Consumers, within the report Advancing Regulatory Science at FDA: Focus Areas of Regulatory Science. This priority advances understanding of ways to engage patients and consumers to better understand the US patients’ and public’s perspectives and preferences related to outcomes [14].

This pilot study explores the potential for social listening data to expand our understanding of its use for gathering patients’ and caregivers’ perspectives. The goal of this project was to gain insights into the key challenges and barriers faced by persons living with chronic pain (PLWCP), how they mitigate or treat chronic pain, perspectives and experience with medication dependence and addiction, and how they describe their pain and measures of success. Utilizing multiple patient perspective data sources offers the FDA the opportunity to capture diverse patient voices and experiences on chronic pain.

## Methods

The Inspire research team regularly compiles and analyzes unstructured patient data to draw out key themes. Over 1,700,000 members have joined Inspire through its website [15], to share their patient journey, ask and answer questions, and engage with other members who know what they are going through by writing posts and responding to others’ posts. These members belong to one or more of over 240 communities focused on specific conditions or disease areas.

To extract relevant posts for this study, predefined keywords and TextRazor tags [16] were identified and used to extract Inspire posts. Harvested posts were published between January 1, 2017, and October 22, 2019, the latter being the date the posts were extracted. The first data set comprised all chronic pain posts that contained #ChronicPain and at least one other relevant disease tag such as #Migraine or #NervePain. The second data set contained #ChronicPain plus a relevant chronic pain management tag or a chronic pain management tag for a treatment or activity specific to chronic pain (eg, #SpinalCordStimulator). Table 1 shows the full set of keywords and TextRazor tags used for harvesting posts. All keywords and tags accounted for misspellings and variations in spelling, and TextRazor tags additionally accounted for synonyms.

**Table 1:** Full set of keywords and TextRazor tags used for harvesting web-based posts.

Category, keywords			TextRazor tags
**Chronic pain^a^**			
	Chronic pain	ChronicPain	
**Common diagnoses related to chronic pain**			
	Back pain	BackPain, LowBackPain	
	Carpal tunnel syndrome	CarpalTunnel, CarpalTunnelSyndrome	
	Complex regional pain syndrome	ComplexRegionalPainSyndrome	
	Failed back surgery syndrome	FailedBackSyndrome	
	Fibromyalgia	Fibromyalgia	
	Migraine	Migraine	
	Multiple sclerosis	MultipleSclerosis	
	Muscle spasms, muscle pain	MusclePain, Spasm	
	Nerve pain	NervePain	
	Neuropathy	NeuropathicPain, PeripheralNeuropathy	
	Peripheral vascular disease	PeripheralArteryDisease	
	Phantom limb pain	PhantomPain	
	Sickle cell disease	SickleCellDisease	
	Spasticity	Spasticity	
	Spinal cord injury	SpinalCordInjury	
	Cerebral palsy	CerebralPalsy	
**Medication-assisted treatment**			
	Buprenorphine, Butrans	Buprenorphine, BuprenorphineNaloxone	
	Dilaudid	Dilaudid	
	Evzio	Evzio	
	Suboxone	Suboxone	
	Fentanyl	Fentanyl	
	Hydrocodone	Hydrocodone	
	Hydromorphone	Hydromorphone	
	Methadone	Methadone	
	Morphine	Morphine	
	Naloxone, Norco	Naloxone, Narcan	
	Naltrexone	Naltrexone, LowDoseNaltrexone	
	Norco	Norco	
	Oxy, oxycodone, oxycontin	Oxycodone	
	Percocet	Percocet	
	Stadol	Butorphanol	
	Vicodin	AcetaminophenHydrocodone	
	Demerol, meperidine	Demerol	
	Prescription pain medication	PainMedication	
	Chronic pain medication	PainMedication	
	Opioid	Opioid, Opiate, OpioidEpidemic	
**Devices**			
	TENS^b^	TranscutaneousElectricalNerveStimulation, Neurostimulation, Transcutaneous electrical nerve stimulationunit	
	Pain pump~^c^	PainPump	
	Drug pump	DrugPump	
	Implantable pump	Implantable pump	
	Opioid pump	Opioid pump	
	Patient-controlled analgesia pump	Patient-controlled analgesia pump	
	Spinal pump~	Spinal pump~	
	PNS^d^	peripheral nerve stimulator, PercutaneousTibialNerveStimulation	
	Spinal cord stimulator~	SpinalCordStimulator	
**Pain management**			
	Pain management	PainManagement	
	Addiction, addicted	Addiction	
	Health care provider, physician, pain specialist	Physician	

^a^This tag was included in all posts other than those focused on chronic pain–specific treatment.

^b^TENS: transcutaneous electrical nerve stimulation.

^c^Tilde (~) indicates posts focused on a chronic pain–specific treatment.

^d^PNS: peripheral nerve stimulator.

The data pull yielded 3156 posts with the following information recorded for each: post title, post content, unique user token of author, time stamp of when the post was published, geographic location of where the post was published, and gender and age per self-report from initial registration or user profile. Posts were subsequently excluded if they did not contain any text (eg, only contained images or videos) or were duplicates of other posts. For the data set about chronic pain management, posts were read to ensure the inclusion of content about management strategies for chronic pain and not only comorbidities.

In-depth analyses were performed on approximately a third of all posts (ie, 920 posts after duplicates and image and video-only posts were removed). Within these posts, approximately half were about chronic pain (494 posts) and the remaining half (426 posts) were about chronic pain management. The 2 data sets—the chronic pain data set and the chronic pain management data set—were examined individually and had different codebooks. The codebooks were developed following a 4-level hierarchy of decision-making: during open coding, text was carefully analyzed from each post to identify preliminary themes (level 1), and then preliminary codes were discussed among the coders (level 2). After reviewing the data, codes were finalized (level 3) and then for better characterization further divided into subcodes (level 4), thereby ensuring a robust model of consensus-based analysis, which means that the final tags did not stem from 1 analyst but 2. In this case, both coders discussed and reached consensus on what the codes and subcodes should be, and then the posts were tagged accordingly. Themes and subthemes were developed using a data-driven approach, relying on a constant comparative method that closely followed that of Osadchiy et al’s [17] social listening study. Inspire’s research team first created a data coding tracker in the targeted Inspire data pull, which identified the overarching topics by which analysts would organize the analysis. Next, analysts created a data codebook, which identified the terms and topics that could be coded under each tracker column for each post. Using this codebook, researchers manually read, analyzed, and tagged each post for key trends and topics. All disagreements were resolved by discussion with team members talking through their coding logic and coming to a consensus.

As seen in an overview of the codebooks (Table 2), the analyses consisted of 3 main parts: (1) lexical analysis, which investigated rhetorical strategies within posts about chronic pain, (2) identification of treatment types and sources for posts about chronic pain management, and (3) content analysis about key challenges and measures of success for both data sets. In order to establish the themes and subthemes for classification, a random sampling of posts was read, characterized, and discussed. Once the categories for coding were agreed upon, posts were reread and all posts subsequently coded.

**Table 2:** Overview of the codebooks.

Codebook, theme		Subtheme
**Disease codebook**		
	Chronic pain lexicon	metaphor/imagery: “severe” scale/level: “flare,” “worsening,” “extreme,” “constant,” “exhausting,” “aching,” “horrible,” “debilitating” other lexicon
	Symptoms	fatigue depression, anxiety irritable bowel syndrome symptoms insomnia nausea, vomiting confusion, brain fog dizziness, vertigo neuropathy other symptoms
	Key challenges	quality of life impact poor disease management lack of diagnosis or misdiagnosis stigma and social impact bad health care provider emotional impact comorbidities lack of support finding health care provider flares limited health literacy impact on loved ones loss of independence/autonomy
	Measures of success	good disease management finding support successful diagnosis improving quality of life finding good health care provider health literacy decreased stigma remission maintaining autonomy
**Treatment codebook**		
	Treatment type	opioid or narcotic device alternative: item alternative: activity anticonvulsant surgery or procedure sedative or anesthetic nonsteroid anti-inflammatory drugs steroid muscle relaxant antidepressant other treatment types
	Specific treatments	oxycodone/oxycontin marijuana/cannabis spinal cord stimulator gabapentin physical therapy diet Lyrica tramadol exercise TENS^a^ other specific treatments
	Mode of administration	oral subcutaneous transcutaneous topical sublingual other mode of administration
	Treatment source	health care provider prescription over the counter or web-based store illegal source friends or family
	Treatment emotions	negative (angry, desperate, afraid…) positive (hopeful, satisfied, grateful…) neutral (cautious, curious, confused…)
	Key challenges and barriers	tolerability, side effects lack of efficacy access: health care provider stigma addiction, dependence access: legal quality of life impact lack of health literacy low dosage difficult administration other challenges
	Measures of success	improving quality of life efficacy access tolerability reducing medications lack of stigma lack of addiction other measures of success

^a^TENS: transcutaneous electrical nerve stimulation.

### Ethical Approval

The New England Independent Review Board and the FDA both approved this study, finding it minimal risk and met the requirements for a waiver of consent (New England IRB# 120190469; the FDA #: 2023-OC-060). The informed consent process was waived for this study because this was secondary data analysis.

## Results

A total of 920 posts by 360 authors who resided in the United States were manually analyzed. When posts contained direct references to a “self” (and the type of self could be determined on the basis of analysis of content), the authors were classified as either patients or caregivers. In posts identifying the author (865/920, 94%), the majority were patients (813/865, 93.9%) followed by caregivers (52/865, 6%). If an author mentioned being both a patient and caregiver, then the author was only classified as the former for the purposes of this research. Per registration and profile data, self-reported gender was collected for 310 (86.1%) of the 360 authors: 89% (276/310) identified as female and 10.9% (34/310) identified as male. During the time of the post extraction, there was no option for nonbinary gender selection on Inspire. Age was also self-reported for 84.4% (304/360) of the authors, with the majority in 40-69 years of age (see Table 3). Overall information on race or ethnicity could not be discerned, as most user profiles lacked such information. This information was not collected on Inspire at the time of the post extraction.

**Table 3:** Age data of the authors (n=304).

Age range (years)	Values, n (%)
18-29	13 (4.3)
30-39	39 (12.8)
40-49	58 (19.1)
50-59	73 (24)
60-69	93 (30.6)
>70	28 (9.2)

The specific diseases and conditions mentioned most frequently in association with chronic pain were fibromyalgia (43/360, 11.9%), Ehlers-Danlos syndrome (33/360, 9.2%), complex regional pain syndrome (19/360, 5.3%), cancer (18/360, 5%), and chronic migraine (18/360, 5%). More than 65 other chronic pain conditions were mentioned less than 5% of the time, including back injury, scleroderma, and rheumatoid arthritis. Nearly half of the authors of posts (162/360, 45%) who mentioned a specific comorbidity also wrote about experiencing multiple comorbidities, with an average of 2.5 conditions mentioned per author on average.

Within the first data set (ie, specific to chronic pain and not its management), the Inspire research team identified 5 rhetorical themes among the posts that contextualized personal experiences of living with chronic pain. The team categorized the 5 themes in this study as subjective scales, examples of quality of life impact, frequency and length of pain descriptors, illustrative characterizations of pain, and self-validating language based on the content (see Table 4). Often a single post contained 2 or more of these themes, and all of them were used to impart information about pain intensity or quality. Moreover, rarely (in <2% of posts) did posts contain mitigating language such as mild, minimal, moderate, tolerable, or stable to describe the chronic pain. When such adjectives or adverbs were used, they were wielded to reflect how authors perceived others such as health care providers’ perspectives of chronic pain.

…Well unfortunately in my area there are no temporomandibular joint dysfunction (TMJ) dysfunction support groups as TMJ is viewed as a mild condition not worthy of even having a support group.

Person with fibromyalgia

**Table 4:** Five rhetorical themes among the posts on personal experiences of living with chronic pain.

Theme	Function	Examples
Subjective scales	Convey how patients feel relative to their baseline levels of chronic pain	“new level of pain” “very severe” “immense” “worsening” “manage my pain level”
Examples of quality of life impact	Show concrete examples of how chronic pain impacts various aspects of life	“disruptive” “disabling” “daily struggle” “barely tolerable”
Frequency and length of pain descriptors	Demonstrate the regularity of chronic pain	“daily” “intermittent” “unceasing “progressive”
Illustrative characterizations of pain	Pinpoints differences in quality of chronic pain experienced	“burning” “throbbing” “radiating” “sharp”
Self-validating language	Emphasizes the authenticity and weight of lived chronic pain experiences	“legitimate” “actual” “real” “serious”

Nearly all published posts about chronic pain contained content about the key challenges (437/494, 88.4%) with impact on quality of life the most frequent challenge mentioned (73/437, 16.7%), with quality of life defined as performing daily activities such as cooking and bathing as well as interacting with others. The full complement of key challenges can be found in Table 5.

**Table 5:** Full complement of key challenges (n=437).

Key challenge	Values, n (%)
Impact on quality of life	73 (16.7)
Managing disease	45 (10.3)
Proper diagnosis	44 (10.1)
Stigma and social impact	39 (8.9)
Relationship with health care providers	33 (7.5)
Emotional impact of disease	31 (7.1)
Navigating comorbidities	27 (6.2)
Lack of support from loved ones	27 (6.2)
Flare-ups	16 (3.7)
Limited health literacy	14 (3.2)
Loss of autonomy	3 (0.7)
Other	85 (19.4)

Approximately 37.8% (187/494) of the posts discussed measures of success for living with chronic pain. The top measures of success within these posts were having good disease management (53/187, 28.3%), maintaining social support (49/187, 26.2%), getting a proper diagnosis (48/187, 25.7%), improving quality of life (47/187, 25.1%), and working with health care providers by willing to listen and advocate for them (47/187, 25.1%). Other measures of success included developing greater health literacy (18/187, 9.6%), noticing less stigma around chronic pain (9/187, 4.8%), being in remission (8/187, 4.3%), and feeling increased autonomy (5/187, 2.7%).

Of the 426 coded posts about chronic pain management, 96.2% (410/426) mentioned a category of chronic pain relief. Opioids or narcotics were mentioned most often (105/410, 25.6%) with oxycodone discussed most frequently (44/105, 41.9%), followed by tramadol (13/105, 12.3%). Only a minority of posts mentioned anticonvulsants (29/410, 7%) such as pregabalin (14/29, 48.3%) or gabapentin (19/29, 65.5%). Few posts mentioned surgery or procedures (24/410, 5.9%) or sedatives or anesthetics (23/410, 5.6%), with lidocaine and acetaminophen equally represented (9/23, 39.1% each). A full accounting of the pain management types can be found in Table 6.

**Table 6:** Pain management type (n=410).

Pain management type	Values, n (%)
Opioids/narcotics	105 (25.6)
Device (eg, spinal cord stimulators, TENS^a^)	56 (13.7)
Alternative substances (eg, cannabis)	48 (11.7)
Alternative interventions (eg, physical therapy, diets, exercise)	44 (10.7)
Anticonvulsants	29 (7.1)
Surgery	24 (5.9)
Sedatives/anesthetics	23 (5.6)
Nonsteroidal anti-inflammatory drugs	20 (4.9)
Steroids	17 (4.1)
Muscle relaxants	14 (3.4)
Antidepressants	13 (3.2)
Other	17 (4.1)

^a^TENS: transcutaneous electrical nerve stimulation.

Treatment sources were reported in 30.7% (142/462) of the posts, with the majority of these indicating that the treatment under discussion was prescribed by a health care provider (103/142, 72.2%). Rarely did posts refer to over-the-counter or web-based vendors (28/142, 19.7%). An even smaller subset of posts mentioned procurement through the street or from friends or family (11/142, 7.7%). When discussing treatment sources, particularly for opioids, posts often made a point to mention having at least at one point a legitimate script from a health care provider.

…When the pharmacy refused to fill a legitimate script, I was left in a very bad way. My husband couldn't stand to watch me suffer; we are law-abiding people, but he was going to buy me something off the street - those ninnys in office don't see they are driving patients with chronic pain to despair.

Person with Ehlers-Danlos syndrome

Challenges about chronic pain management were mentioned in 38.5% (164/426) of the posts. The 2 most frequent challenges discussed in posts were tolerability (55/164, 33.5%) and lack of efficacy (54/164, 32.9%). Nearly a third of the posts mentioned difficulty accessing treatments from health care providers (49/164, 29.9%); a smaller number of posts mentioned stigma around their condition (27/164, 16.5%) and addiction (28/164, 17.1%). Some posts featured challenges such as legal access (20/164, 12.2%), low health literacy (18/164, 10.9%), quality of life impact (16/164, 9.8%), low dosage (15/164, 9.1%), difficulty with administration (14/164, 8.5%), and other challenges (15/164, 9.1%), including cost and time. When broaching these challenges, many of the posts were contextualized within the opioid crisis. Overall, authors seemed conflicted, recognizing that long-term opioid usage leads to dependence but also feeling exasperated by not being able to find other treatments with similar levels of pain relief.

…Until recently, I took more medication, and was able to function better. However, I can see the pressure my rheumatologist is under to limit the prescription of narcotics, and I do not want to cause problems for her. This makes me sad, because I am in constant pain and my mobility and quality of life are severely affected. A couple of days ago, I broke down in tears because I was in so much pain, yet so conflicted about taking more medication; sometimes, I feel quite downhearted about it.

Person with Ehlers-Danlos syndrome

Measures of success for chronic pain management were featured in approximately a third of posts (137/426, 32.2%). Within posts, measures of success included improved quality of life (87/137, 63.5%) and efficacy (86/137, 62.8%). Posts that mentioned personal experience with opioids often stated improved quality of life as the primary reason they preferred or were grateful for opioids.

…I also had 10 opioid pills. The second day home a pain came suddenly to my gut and chest area. It was late on a Friday night. A stabbing pain, more intense than any I have ever had. Thank god for the pain medication. [...] the difference between screaming in pain and resting somewhat comfortably is not something I would want to live without.

Person with Ehlers-Danlos syndrome and cancer

Other measures of success were access to chronic pain management (27/137, 19.7%) and tolerability of the management (26/137, 18.9%). Less commonly mentioned were reduction of medications or dosages (12/137, 8.8%), decreased stigma (11/137, 8%), not being dependent or addicted (8/137, 5.8%), and other measures (11/137, 8%) such as ease of administration, health literacy, and compliance.

## Discussion

This study explores the potential for utilizing social listening data to expand our understanding of its use for gathering patients’ and caregivers’ perspectives of chronic pain. It is important to understand user-generated content about chronic pain and chronic pain management from social media and web-based peer-to-peer health networks. In addition, key challenges and barriers faced by PLWCP as well as how they mitigate or treat chronic pain were identified from these platforms. For example, there were some key differences in chronic pain discussions between general social media and peer-to-peer health networks. In general, research has documented that social media sites (eg, Reddit, Instagram, Tumblr, Pinterest, Twitter) act as venues for patients seeking others’ advice and stages from which to legitimize their experiences and build empathy [18,19]. In this way, digital conversations and narratives help make invisible chronic pain visible and combat the culture of disbelief, that is, the failure to accept an individual’s account of his or her pain as true [20-22]. On networks such as Inspire, the audience within the venue is more targeted and includes only other patients, caregivers, and the occasional health care provider. Yet, even in this relatively safe environment, we found that authors of chronic pain habitually felt the need to use rhetorical appeals to ground and situationalize their questions and advice. This may, in part, reflect the extent to which the culture of disbelief is internalized by patients and caregivers and impacts their chronic pain experiences.

It is in this context that posts about relief for chronic pain also exist. Studies within health care spaces have revealed that patients felt disrespected and suspected of drug-seeking when seeking chronic pain management even before the height of the opioid crisis [23]. Part of the issue may be differences in patients’ and health care providers’ relative priorities for pain management. Patients’ top priorities are generally reduction of pain intensity, followed by diagnosing the cause of the pain, whereas health care providers’ top priorities are generally improving function, followed by reducing medication side effects [24]. Approximately 24.6% (105/426) of the chronic pain management posts from Inspire mentioned opioids or narcotics. Although there is awareness within these posts that the long-term regular usage of opioids can lead to dependence and that misuse of opioids is common, it is important to note that many PLWCP either (1) do not consider themselves at risk for addiction or (2) consider this risk less important than immediate relief from pain. This matches what other studies have found, with the reasoning there being that patients tended to regard themselves as exceptions since they were genuinely in pain and were not engaging in aberrant behaviors such as asking for early refills or taking more medications than prescribed [25].

Patients are keenly aware of the stigma surrounding opioids or narcotics and crave other efficacious management strategies, which can be seen in the language they use within their posts. PLWCP who mention using opioids in Inspire posts frequently assert that they take the “lowest possible dose” or that this is the “only treatment which has been successful” or that they “only take the medication as needed.” When compared to opioids or narcotics, other chronic pain management strategies tended to be positioned as ineffective. For instance, when marijuana or gabapentin was mentioned in posts, these treatments were portrayed as unsuccessful as compared to the immediate and long-lasting relief of opioids. Even so, some PLWCP reported moderate success with anticonvulsants and sedatives, although both anticonvulsants and sedatives were mentioned less than 30 times each, and these results should be taken with caution. Similarly, there appears to be increasing awareness that medical devices such as spinal cord stimulators and TENS (transcutaneous electrical nerve stimulation) machines may help alleviate chronic pain. Patients without personal exposure to such devices expressed hope and curiosity about them, actively seeking out personal anecdotes of PLWCP.

To adequately address chronic pain, we need to have a greater awareness of the multifaceted discussions that PLWCP are having online, particularly on digital peer-to-peer health networks. As seen in the key challenges mentioned in Inspire posts, many PLWCP felt as though they exposed themselves to social and institutional barriers that have made them feel even more vulnerable and isolated than before when attempting to reduce pain intensity. Nearly a third of the posts about chronic pain management mentioned difficulty accessing treatments from health care providers (49/164, 29.9%), followed by stigma (27/164, 20.7%). Even those who did not mention chronic pain management in their posts reported stigma and social impact (39/437, 8.9%) and having poor relations with health care providers (33/437, 7.6%). As other studies have documented, the health care system has not always been structured to reflect a continuum of care for pain, resulting in barriers that can impede persons with chronic pain from receiving timely access to care [26]. Analysis of web-based conversations, especially those directed to and for other patients and caregivers, should inform how we attempt to address chronic pain barriers and measures of success. Particularly important is better understanding patient and caregiver perceptions of the available treatment options and what approaches might encourage them to try management strategies that have a low risk of dependency.

The findings in our report are subject to several limitations. First, because of the digital divide, those who post on web-based peer-to-peer health networks are not representative of the general population. Although this is beginning to change in the age of mobile-friendly websites, this still means that those who are unable to afford a mobile device or have easy access to Wi-Fi are limited in their ability to participate in these networks. Second, this study had a relatively small sample of posts mentioning anticonvulsants, sedatives, and treatment devices for chronic pain. Future studies should further investigate patient perspectives of these chronic pain management strategies, as this literature is still in its infancy. The 5 themes in our study did not have any theoretical framework to support the rhetoric or related research fields, which is a limitation. Researchers have become increasingly interested in the social context of chronic pain conditions, including pain severity, physical disability, pain behaviors, and psychological distress, and have developed theoretical models [27]. In the future, theoretical models should be incorporated to support analysis of constructs. Another limitation was that only 1 source of data was used for the analysis, which was Inspire-only data. Future studies should expand data sources to include additional social media platforms. Finally, while anonymity is a valuable benefit to participating in a web-based peer-to-peer health network, it also creates difficulties when systematically analyzing user-generated content. Key demographics in this study such as gender and age could not be determined unless patients chose to self-identify upon registration or later via their profile pages. Further, demographic information about race and ethnicity was not collected originally at the time of platform registration, thereby severely limiting the analysis of these characteristics. Recognition of this limitation spurred Inspire to collect race and ethnicity data from new members, thereby improving opportunities for health equity research across their platforms. Additionally, it is important to consider that although the use of social media by patients for health-related reasons is growing rapidly, not all social media platforms are ideal or may appeal to all patients. This study only examined 1 condition on 1 online health community platform, that is, Inspire. Future studies should incorporate other diseases and web-based platforms to gather a more comprehensive understanding [11,28,29]. Lastly, studies should include other potential stakeholders such as family members and health professionals to understand their perspectives on chronic pain management.

This study underscores the role of user-generated content in web-based peer-to-peer health networks to help the health care community better understand the treatment and management experience of some patients with chronic pain. Our results suggest that these conversations could help inform our conceptualization of chronic pain challenges and measures of success, which is especially crucial to capture, considering the culture of disbelief. The rhetorical strategies used in posts on Inspire indicate the extent to which this culture impacts even content written to others with akin experiences. PLWCP are aware of the stigma surrounding certain chronic pain treatments options and crave efficacious management strategies; yet, authors of posts perceived strategies other than opioids to be less effective for substantial long-term relief. Even so, some PLWCP reported moderate success with anticonvulsants and sedatives, and some PLWCP appear to be aware that medical devices such as spinal cord stimulators and TENS machines may help alleviate chronic pain. More analysis is needed of the multifaceted discussions that PLWCP are having with each other online. Particularly important is better understanding patient and caregiver perceptions of relief with available chronic pain methods and what may encourage patients to try strategies that can be safely used to manage chronic pain over long periods of time.

## Abbreviations

FDA: Food and Drug Administration
OMHHE: Office of Minority Health and Health Equity
PLWCP: persons living with chronic pain
TENS: transcutaneous electrical nerve stimulation
